# Supplementary material for: Boosting superconductivity in ultrathin YBa2Cu3O7−δ films via nanofaceted substrates
Source: Nat Commun. 2026 Jan 7;17:285. doi: 10.1038/s41467-025-67500-2 (PMC12783791; doi:10.1038/s41467-025-67500-2)
Supplement: Supplementary file 1 — Supplementary Information [file 41467_2025_67500_MOESM1_ESM.pdf]

Supplementary Information for

Boosting Superconductivity in ultrathin $YBa_2Cu_3O_{7-\delta}$ films via nanofaceted substrates

A. Estimation of doping from c-axis length and critical temperature

To construct the doping–temperature $T(p)$ phase diagram, it is necessary to determine the hole doping level p for each film.

Accurately determining p in YBCO thin films is particularly challenging. On the one hand, the oxygen content n cannot be directly measured, as it can in bulk single crystals via chemical and thermogravimetric analyses [1]. On the other hand, copper in YBCO exists in both Cu^{1+} and Cu^{2+} oxidation states, with holes distributed between these two sites. The hole distribution depends not only on the total oxygen content but also on the degree of oxygen ordering within the CuO chains.

Due to these challenges, an empirical relationship is commonly employed to estimate p from the superconducting critical temperature T_c. This relation, originally established from studies on LSCO, takes the form:

$$1 - \frac{T_c}{T_c^{\max}} = 82.6 \cdot (p - 0.16)^2, \tag{S1}$$

where $T_c^{\max}$ is the maximum critical temperature at optimal doping. This expression provides a reasonable estimate of the hole doping level in most superconducting cuprates, including YBCO, except near $p = 1/8$, where charge ordering suppresses T_c to a degree that depends strongly on the specific compound and, as shown in our manuscript, on strain [2, 3]. This empirical parabolic dependence defines the shaded region in Fig. 2 of the main text.

In our work, we use this relation to estimate p from the measured T_c. As shown in Ref. [3], by combining the knowledge of the length of c-axis lattice parameter with the T_c-based doping estimate derived from the parabolic relationship, we obtain a very robust and consistent method for determining the hole concentration across our series of strained and unstrained films.

B. Estimation of doping from the pseudogap temperature

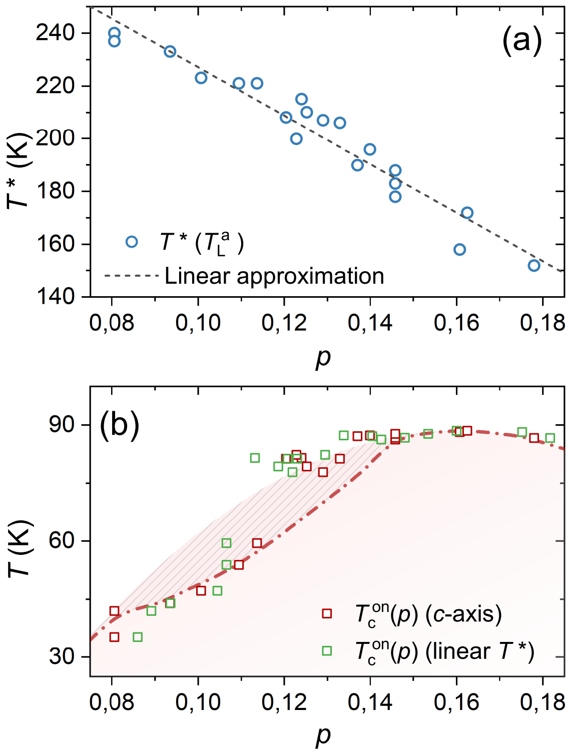

FIG. S1. **Doping estimation using** T*****(a) Linear approximation of the pseudogap temperature T^* versus doping (black line) and T^* extracted from the $R(T)$ of 10 nm thick YBCO films of different doping (blue circles). (b) Comparison of doping dependence of T_c^{on} determined by the c-axis length as described in the main text (red squares) and determined according to the value of T^* (green squares).

As an extra check on the determination of the doping level in the films we can use the pseudogap temperature T^*. It is well known that the pseudogap line in the YBCO phase diagram is linear in doping [4, 5]. Figure S1(a) shows T^* (estimated from T_L^a as described in the main text) of a set of 10 nm thick YBCO films with different doping levels. Clearly, T^* is close to linear in the full doping range. In Figure S1(b) we compare the doping dependence of T_c^{on} estimated from the c-axis length (as described in the main text) and by setting the doping according to the value of T^* by adjusting the level to the linear approximation in Figure S1(a). There is a slight shift of the doping of each film, but the general picture is the same as for the c-axis method. The conclusion is that in addition to the conventional

method using the c-axis length, also T^* is a good measure of the doping level in YBCO.

C. Comparison of the resistive transition of YBCO thin films grown on MgO and STO substrates

In the main text we describe how the resistive transition is modified in underdoped d =10 nm YBCO thin films grown on MgO. We find that both the onset of superconductivity T_c^{on} and the width of the transition in temperature ΔT_c is greatly enhanced compared to thicker (d =50 nm) films. To strengthen that the increased ΔT_c is due to reduced superfluid stiffness, we compare these findings with resistivity measurements from an untwinned d =10 nm YBCO film with similar doping level grown on vicinal angle cut $SrTiO_3$ (STO) (see Figure S2 (c)). Notice that T_c^{on} of the d =10 nm film on STO is close to T_c^{on} of the d =50 nm film on MgO. This is expected since the CDW order in the STO film is not suppressed [5].

The broadening of the resistive transition can be quantified by the full width half maximum of the peak in $d\rho/dT$ at the transition temperature, see Figure S2(d). The width of the resistive transition of the d = 10 nm film on MgO ($\Delta T_c \approx 23$ K) is approximately double compared to that of the d = 10 nm film on STO ($\Delta T_c \approx 13$ K). This is a surprising result since ΔT_c is enhanced in the doping range where CDWs are present in YBCO. One would expect that the broadening of the transition in the film on MgO would decrease, not increase. A possible explanation of the broader transition could be a much larger disorder in the film on MgO, but since the resistivity at room temperature (a/b average) $\rho_{s,av}$ is very close to that of the 10 nm film on STO, see Figure S2(b,c), we can discard this possibility. Disorder can however possibly explain the difference in ΔT_c of the film on STO and the d = 50 nm film on MgO. We therefore conclude that a more likely explanation of the increased broadening comes from a reduced superfluid stiffness.

D. Evaluation of the stiffness for the nematic system

In the model developed in Ref. [6] the electronic states of the cuprate film are hybridized to the elongated facet on the substrate having under-coordinated atoms. The virtual hopping ($\sim t_\perp$) of charge carriers to the substrate atoms (on-site potential of the substrate atoms

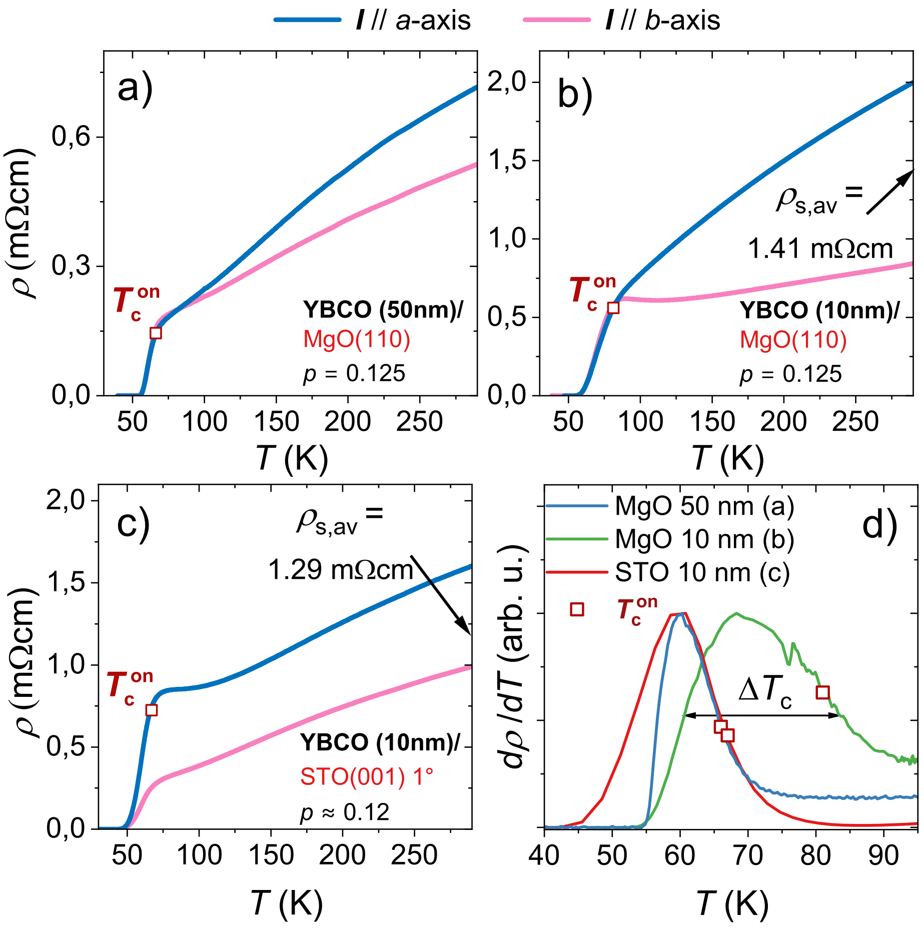

FIG. S2. **Comparison of the resistive transition in underdoped YBCO thin films.**(a) 50 nm thick YBCO on MgO. (b) 10 nm thick YBCO on MgO (c) 10 nm thick YBCO on STO (d) temperature derivative of the resistivity close to the transition for the films in (a,b,c).

V_{sub}) induces an effective repulsion between the atomic levels of film and substrate $V_0 = \frac{1}{2}(\sqrt{V_{sub} + 4t_\perp^2} - V_{sub})$. The strips of atoms (belonging to the facets) which couple to the cuprate film via $t_\perp$ are randomly distributed on the lattice. While in Ref. [6] we have treated this randomness within the coherent-potential approximation [7], in our present treatment we solve the model exactly on finite lattices (up to 40×40 sites) and average over different strip realizations.

As has been argued in Ref. [6], the effective potential for the STO substrate is significantly

smaller than in case of MgO. The effective model reads

$$H = \sum_{ij,\sigma} t_{ij} c^\dagger_{i,\sigma} c_{j,\sigma} + V_0 \sum_n \sum_{s=1}^{L} c^\dagger_{\mathbf{R}_n+s\mathbf{b}} c_{\mathbf{R}_n+s\mathbf{b}} + J \sum_{\langle ij \rangle} \left[\mathbf{S}_i \mathbf{S}_j - \frac{1}{4} n_i n_j \right] \tag{S2}$$

where R_n denotes the starting sites of the 1-D strips with length L along $\mathbf{b}$ and effective potentials V_{eff}. We include nearest ($\sim t$) and next-nearest neighbor ($\sim t'$) hopping parameters and approximate the coupled regions as single rows of sites with $L \to \infty$ which are randomly distributed over the system (concentration c). The d-wave superconductivity is implemented via the decoupling of the spin-spin interaction $\sim J$ which yields a bond order SC order parameter

$$\Delta_{i,\delta} = -\frac{J}{2} \left[\langle c_{i,\downarrow} c_{i+\delta,\uparrow} \rangle + \langle c_{i+\delta,\downarrow} c_{i,\uparrow} \rangle \right] \tag{S3}$$

with $\delta \equiv \pm a, \pm b$. An electromagnetic field along the δ-direction is coupled via the Peierls Substitution $c^\dagger_{n+\delta,\sigma} c_{n\sigma} \to e^{ieA_\delta(n,t)} c^\dagger_{n+\delta,\sigma} c_{n\sigma}$ and we compute the paramagnetic current response along δ from

$$j_p^\delta(n,t) = \sum_m \int dt' \Lambda^{jj}_{\delta\delta}(n,m,t-t') A_\delta(m,t') \tag{S4}$$

with the current-current correlation function

$$\Lambda^{jj}_{\delta\delta}(n,m,t-t') = i \int_{-\infty}^{\infty} dt \Theta(t-t') \times e^{i\omega(t-t')} \left\langle \left[j_p^\delta(n,t), j_p^\delta(m,t') \right] \right\rangle .$$

Finally, the optical conductivity along the δ-direction is obtained from

$$\sigma_{\delta\delta}(\omega) = \frac{e^2}{N} \sum_{n,m} \frac{\Lambda^{jj}_{\delta\delta}(n,m,\omega) + \delta_{n,m} k_\delta(n)}{i(\omega + i\eta)} ,$$

and $k_\delta(n)$ denotes the kinetic energy on the bond $(n, n+\delta)$. Results are averaged over 10 randomly chosen strip configurations.

Fig. S3 reports the real and imaginary parts of the optical conductivity for the same parameters as in Ref. [6] used to characterize the electronic structure of cuprate films on MgO and STO, respectively. Since both, facets on MgO and step edges on STO, run along the orthorhombic b-direction the optical conductivity along both directions is vanishingly small

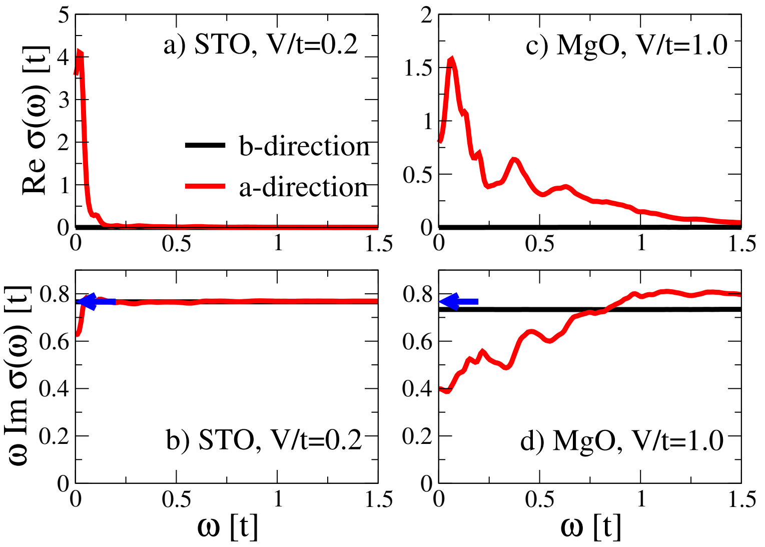

FIG. S3. **Optical conductivity:** comparison between MgO and STO substrates **a,c,** Real part of the optical conductivity for MgO and STO, respectively. (**b,d**) Imaginary part multiplied by frequency for MgO and STO, respectively. Red (black) curves refer to the orthorhombic a (b) direction and the stiffness of the homogeneous system is indicated by the blue arrow. The results have been obtained by averaging over 10 disorder configurations. Further parameters: $t/t' = -0.15$, doping $p = 0.12$, $J/t = 1$, concentration of strips $c = 0.05$.

in our model, since the associated strips are taken as infinitely long so that the system appears homogeneous along b. In contrast, the inhomogeneity perpendicular to the facets/step edges produces a finite absorption which for the case of MgO (panel c) is extended to significantly higher frequencies. From Kramers-Kronig this implies stronger reduction of the imaginary part at small frequencies. Since the stiffness is related to the imaginary part via $D_s = \omega Im\sigma(\omega)$ one therefore observes a reduction of the stiffness along the a-direction by almost 50% for the MgO parameters whereas the reduction is only 15% in case of STO.

E. Vortex melting model to estimate $H_{c,2}$

Previously, the full doping dependence of $H_{c,2}$ in YBCO single crystals has been extracted by extrapolating the vortex lattice melting field to zero temperature [8]. In strongly anisotropic type-II superconductors (like YBCO) the vortex lattice melts at the magnetic field B_M that is less than $H_{c,2}$ (for $T \neq 0$ and $T \neq T_c$) causing a finite resistance. According

to theory

$$\frac{\sqrt{b_m(t)}}{1-b_m(t)}\frac{t}{\sqrt{1-t}}\left(\frac{4(\sqrt{2}-1)}{\sqrt{1-b_m(t)}}+1\right)=\frac{2\pi c_L^2}{\sqrt{Gi}} \tag{S5}$$

Where $b_m = B_m/\mu_0 H_{c,2}$ is the reduced melting field, $t = T/T_c$ is the reduced temperature, c_L a constant (Lindemann number) and Gi the Ginzburg number $Gi \approx (9.225 \cdot 10^8 \mu_0 H_{c,2} T_c \lambda_{ab} \lambda_c)^2$, where λ_{ab} and λ_c are the zero temperature in-plane and out-of-plane London penetration depth respectively [9].

In the previous publication Ramshaw et. al. used equation S5 to estimate $H_{c,2}$ from measurements of $b_m(t)$ by using known values of λ_{ab} and λ_c. Since the doping dependence of these parameters is unknown in our thin films, we fix $H_{c,2}$ to the value estimated by the linear fits as described in the main text and keep $c_L^2/\lambda_{ab}\lambda_c$ as a free parameter. B_m is defined as the field where the resistance reaches 1% of the value at 55 T. Figure S4 (a,b) shows the results of the fitting. For the 50 nm thick film we get $c_L^2/\lambda_{ab}\lambda_c = 2.8\ \mu\text{m}^{-2}$ and for the 10 nm thick film $c_L^2/\lambda_{ab}\lambda_c = 1.9\ \mu\text{m}^{-2}$. Both these numbers are smaller than what was estimated for single crystals [8], which is expected since the penetration depth λ increases when going from bulk to thinner films. A larger number for the 50 nm thick film is also

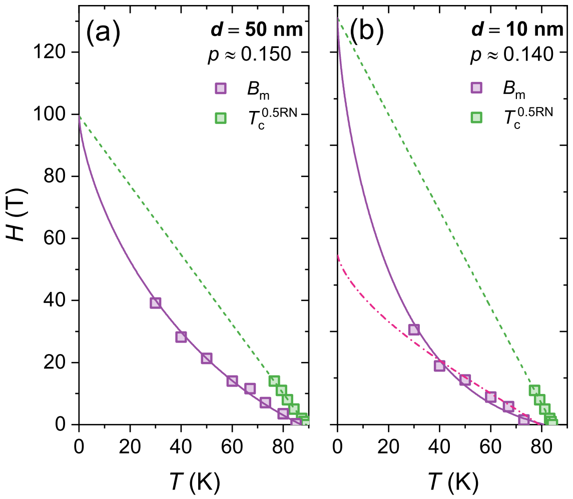

FIG. S4. **Vortex lattice melting fits to estimate $H_{c,2}$ in YBCO thin films** (a) d =50 nm, $p = 0.15$ (b) d =10 nm, $p = 0.14$. The dashed lines are linear fits of $T_c^{0.5R_N}$ and the full lines vortex lattice melting fits of B_m. The pink dash-dotted line in (b) is a vortex lattice melting fit of B_m with $H_{c,2}$=55 T.

expected because of the higher doping level which decreases λ. Since we want to use the vortex lattice melting fitting to provide additional proof of the enhancement of $H_{c,2}$ in the $d = 10$ nm films we also made a fit using the expected $H_{c,2}$ value (as in single crystals and thicker films) at $p = 0.14$ which is 55 T, see the pink dash-dotted line in figure S4(b). The resulting fitting gets a curvature sign that changes with temperature which is not expected for a melting line in YBCO and deviates. In addition it sensibly deviates from the data at low temperature. We therefore conclude that a lower critical fields close to $H_{c,2}$ of the bulk, are not compatible with the vortex melting scenario for the data on 10 nm thick thin films on MgO substrates.

F. Implementation of magnetic field and solution of gap equations

The external magnetic field is implemented via the Peierls substitution which introduces a phase factor to the hopping amplitude as $t_{ij}(A) = t_{ij}\exp\{(ie/\hbar c)\int_{r_i}^{r_j} A.dl\}$. Here A is the vector potential corresponding to the applied magnetic field H and we choose to work in the Landau gauge, $A = H(0, x, 0)$. Furthermore, it is convenient to introduce the reduced dimensionless magnetic field $h = eH/(\hbar c)$. Note that we do not consider the Zeeman effect of the magnetic field, since the Pauli limit for cuprate superconductors is very large [10], i.e., the orbital effect of the magnetic field will destroy superconductivity by creating vortices at fields where the Zeeman effect can still be neglected.

The upper critical field $h_{c,2}$ is obtained by determining the field at which the superconducting order parameter Δ_{ij} becomes zero. This can be accomplished by solving the linearized gap equation, which is valid near both the critical transition field or temperature [11, 12],

$$\Delta_j^{\delta} = -\frac{J}{2}\sum_{i,\delta'} M_{ji}^{\delta'\delta}\Delta_i^{\delta}\,. \tag{S6}$$

Here $\delta = +a, +b$ represents nearest-neighbor bonds and

$$\begin{aligned} M_{ji}^{\delta'\delta} &= \sum_{mn}\left(u_{jn}u_{j+\delta' m} + u_{j+\delta' n}u_{jm}\right) \\ &\times\left(u_{im}^{*}u_{i+\delta n}^{*} + u_{i+\delta m}^{*}u_{in}^{*}\right) \\ &\times\frac{\tanh(E_m/2k_BT) + \tanh(E_n/2k_BT)}{2\,(E_m + E_n)}, \end{aligned} \tag{S7}$$

where u_{im} are the eigenvectors corresponding to the eigenvalues E_m of the non-superconducting

part of the Hamiltonian, $H_0 + H_{\rm CDW}$ in Eq. (2) of the main text, and T is the temperature. The linearized gap equation Eq. (S6) admits non-zero solutions, and thus a superconducting state, for Δ_j^δ if $\det(M - I) = 0$ with

$$M = \begin{pmatrix} M^{aa} & M^{ab} \\ M^{ba} & M^{bb} \end{pmatrix}, \tag{S8}$$

where I is an $2N \times 2N$ identity matrix and $M^{\delta'\delta}$, given by Eq. (S7), are $N \times N$ matrices with N the system size. The solution $\det(M - I) = 0$ corresponds to finding the conditions for which the largest eigenvalue of the matrix M is unity, with the corresponding temperature T_c and magnetic field $h_{c,2}$ marking the transition into the superconducting state. T_c and $h_{c,2}$ are then calculated for two different models discussed in the main text mimicking the 10 nm and 50 nm films. The pairing interaction J is kept the same for both models with different nematicity α. This is inspired by the following fact. From the strong coupling perspective, the pairing interaction is given by $J = 4t^2/U$ where t is the hopping integral and U is the energy cost for onsite double occupancy. In nematic system, it becomes anisotropic with $J_a = 4t_a^2(1-\alpha)^2/U$ and $J_b = 4t_b^2(1+\alpha)^2/U$, where we have used the same parametrization of the hopping integrals as in the main text. For the nematic system, we then find that inclusion of the anisotropy in the pairing interaction changes the dominant d-wave order parameter by less than 1%. Hence, we take the same value of J for different α in obtaining the phase diagram in Fig. 5 of the main text.

G. Explanation of selected CDW vectors.

In our theoretical analysis we approximate the two-dimensional CDW scattering vectors observed by RIXS in the 50 nm films by a checkerboard modulation with $Q^{cdw} = (0.5, 0.5)$ [r.l.u.]. Figure S5(a) demonstrates that the observed CDW (red arrows) corresponds to four hot spots in each quadrant of the Brillouin zone at where the d-wave SC gap is of intermediate size. The theoretically implemented CDW (blue arrow) replaces these four scattering points by two hot spots in the antinodal region where the d-wave gap is large which decrease the computational complexity and make the problem numerically solvable. Thus, in both cases the CDW scattering has a similar and significant impact on the SC state, as evident by the same substantial decrease in T_c.

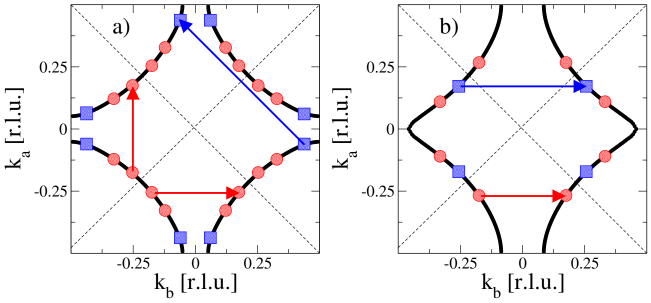

FIG. S5. **CDW: comparison between selected vectors and RIXS measurements. a,b,** The hot spots of the CDW (blue squares) implemented in "Model 1" (**a**) and "Model 2" (**b**) are shown, together with the CDW measured in RIXS (red circles). Note that the hot spots include Umkplapp scattering. Dashed lines indicate the nodes of the d-wave gap.

In case of the 10 nm films (Figure S5(b)), the implemented model 2 substitutes the observed one-dimensional scattering along the b-direction with $|Q^{cdw}| = 0.3$ (red arrow) by a uni-axial scattering along the same direction but with larger magnitude $|Q^{cdw}| = 0.5$ (blue arrow) to mimic the same effect on T_c. Including Umklapp scattering, the number of hot spots in each quadrant is reduced from 2 for the experimental Q^{cdw} to 1 for the theoretical model. It is therefore expected that the latter somehow underestimates the influence of the CDW on T_c. However, the result that model 2 has a smaller impact on T_c as compared to model 1 is still expected to hold due to the reduced number of nesting points.

---

[1] R. Beyers, B. T. Ahn, G. Gorman, V. Lee, S. Parkin, M. Ramirez, K. Roche, J. Vazquez, T. Gür, and R. Huggins, Oxygen ordering, phase separation and the 60-k and 90-k plateaus in yba2cu3ox, Nature **340**, 619 (1989).

[2] J. Torrance, A. Bezinge, A. Nazzal, T. Huang, S. Parkin, D. Keane, S. LaPlaca, P. Horn, and G. Held, Properties that change as superconductivity disappears at high-doping concentrations in la2-xsrxcuo4, Phys. Rev. B **40**, 8872 (1989).

[3] R. Liang, D. Bonn, and W. Hardy, Evaluation of cuo2 plane hole doping in yba2cu3o6+x single crystals, Phys. Rev. B **73**, 180505 (2006).

[4] N. Barišić, M. K. Chan, Y. Li, G. Yu, X. Zhao, M. Dressel, A. Smontara, and M. Greven, Universal sheet resistance and revised phase diagram of the cuprate high-temperature super-

conductors, Proc. Natl. Acad. Sci. U.S.A. **110**, 12235 (2013).

[5] E. Wahlberg, R. Arpaia, G. Seibold, M. Rossi, R. Fumagalli, E. Trabaldo, N. B. Brookes, L. Braicovich, S. Caprara, U. Gran, *et al.*, Restored strange metal phase through suppression of charge density waves in underdoped $YBa_2Cu_3O_{7-\delta}$, Science **373**, 1506 (2021).

[6] G. Mirarchi, R. Arpaia, E. Wahlberg, T. Bauch, A. Kalaboukhov, S. Caprara, C. Di Castro, M. Grilli, F. Lombardi, and G. Seibold, Tuning the ground state of cuprate superconducting thin films by nanofaceted substrates, Commun. Mater. **5**, 146 (2024).

[7] B. Velickỳ, S. Kirkpatrick, and H. Ehrenreich, Single-site approximations in the electronic theory of simple binary alloys, Phys. Rev. **175**, 747 (1968).

[8] B. Ramshaw, J. Day, B. Vignolle, D. LeBoeuf, P. Dosanjh, C. Proust, L. Taillefer, R. Liang, W. Hardy, and D. Bonn, Vortex lattice melting and $H_{c,2}$ in underdoped $YBa_2Cu_3O_y$, Phys. Rev. B **86**, 174501 (2012).

[9] G. Blatter, M. V. Feigel'man, V. B. Geshkenbein, A. I. Larkin, and V. M. Vinokur, Vortices in high-temperature superconductors, Rev. Mod. Phys. **66**, 1125 (1994).

[10] A. S. Dzurak, B. E. Kane, R. G. Clark, N. E. Lumpkin, J. O'Brien, G. R. Facer, R. P. Starrett, A. Skougarevsky, H. Nakagawa, N. Miura, Y. Enomoto, D. G. Rickel, J. D. Goettee, L. J. Campbell, C. M. Fowler, C. Mielke, J. C. King, W. D. Zerwekh, D. Clark, B. D. Bartram, A. I. Bykov, O. M. Tatsenko, V. V. Platonov, E. E. Mitchell, J. Herrmann, and K.-H. Müller, Transport measurements of in-plane critical fields in $YBa_2Cu_3O_{7-\delta}$ to 300 T, Phys. Rev. B **57**, R14084 (1998).

[11] M. Mierzejewski and M. M. Maśka, Upper critical field for electrons in a two-dimensional lattice, Phys. Rev. B **60**, 6300 (1999).

[12] M. M. Maśka and M. Mierzejewski, Upper critical field for anisotropic superconductivity: A tight-binding approach, Phys. Rev. B **64**, 064501 (2001).
